# Network Pharmacology for Analyzing the Key Targets and Potential Mechanism of Wogonin in Gliomas

**DOI:** 10.3389/fphar.2021.646187

**Published:** 2021-04-07

**Authors:** Zaizhong Wang, Lulu Cheng, Zhigang Shang, Zhihui Li, Yuping Zhao, Wenwen Jin, Yingyue Li, Fangchu Su, Xiaobo Mao, Chuanliang Chen, Jianhua Zhang

**Affiliations:** ^1^ Medical Engineering Technology and Data Mining Institute, Zhengzhou University, Zhengzhou, China; ^2^ Department of Neurosurgery, Zhumadian Central Hospital, Zhumadian, China; ^3^ Digital Medical Laboratory, Zhumadian Central Hospital, Zhumadian, China; ^4^ School of Electrical Engineering, Zhengzhou University, Zhengzhou, China; ^5^ Technology Department, China Academy of Chinese Medical Sciences, Beijing, China; ^6^ Clinical Bioinformatics Experimental Center, Henan Provincial People’s Hospital, Zhengzhou, China

**Keywords:** glioma, wogonin, network pharmacology, signal path, key target

## Abstract

**Objective:** To analyze the key targets and potential mechanisms underlying the volatile components of *Scutellaria baicalensis* Georgi acting on gliomas through network pharmacology combined with biological experiments.

**Methods:** We have extracted the volatile components of *Scutellaria baicalensis* by gas chromatography-mass spectrometry (GC-MS) and determined the active components related to the onset and development of gliomas by combining the results with the data from the Traditional Chinese Medicine Systems Pharmacology database. We screened the same targets for the extracted active components and gliomas through network pharmacology and then constructed a protein-protein interaction network. Using a Gene Ontology and Kyoto Encyclopedia of Genes and Genomes (KEGG) analysis, we analyzed the protein effects and regulatory pathways of the common targets. Lastly, we employed ELISA and Western blot in verifying the key targets in the regulatory pathway.

**Results:** We ultimately determined that the active component in *S. baicalensis* Georgi related to the onset and development of gliomas was Wogonin. The results of the network pharmacology revealed 85 targets for glioma and Wogonin. We used gene ontology to analyze these target genes and found that they involved 30 functions, such as phosphatidylinositol phosphokinase activation, while the KEGG analysis showed that there were 10 regulatory pathways involved. Through the following analysis, we found that most of the key target genes are distributed in the PI3K-Akt and interleukin 17 signaling pathways. We then cultured U251 glioma cells for the experiments. Compared with the control group, no significant change was noted in the caspase-3 expression; however, cleaved caspase-3 expression increased significantly and was dose-dependent on Wogonin. The expression of Bad and Bcl-2 with 25 μM of Wogonin has remained unchanged, but when the Wogonin dose was increased to 100 μM, the expression of Bad and Bcl-2 was noted to change significantly (Bad was significantly upregulated, while Bcl-2 was significantly downregulated) and was dose-dependent on Wogonin. The ELISA results showed that, compared with the control group, the secretion of tumor necrosis factor alpha, IL-1β, and IL-6 decreased as the Wogonin concentration increased. Tumor necrosis factor alpha downregulation had no significant dose-dependent effect on Wogonin, the inhibitory effect of 25 μM of Wogonin on IL-6 was not significant, and IL-1β downregulation had a significant dose-dependent effect on Wogonin.

**Conclusion:** Wogonin might promote the apoptosis of glioma cells by upregulating proapoptotic factors, downregulating antiapoptotic factors, and inhibiting the inflammatory response, thereby inhibiting glioma progression.

## Introduction

Glioblastoma multiforme, also called glioma, is a relatively common malignant brain tumor in adults, which originates from glial cells, is a highly invasive and fatal tumor of the central nervous system (Galldiks et al., 2017), and it is not only prone to relapse but also has a high mortality rate. Relevant studies have shown that the mean survival time for glioma is 6–14 months, which represents a serious threat to the life and health of such patients (Aquino et al., 2017; Wesseling and Capper, 2018). At present, surgical resection combined with postoperative chemoradiotherapy is the main treatment for glioma. Due to issues such as the immunosuppression caused by surgery and chemoradiotherapy, residual tumor cells can grow rapidly in a short period and thereby cause a relapse (Hervey-Jumper and Berger, 2019). Some patients also develop resistance to chemotherapy drugs when taken long term, thereby reducing their therapeutic effect.

Natural products are an abundant treasure trove for the study of and search for active drugs against glioma and other cancers. The use of traditional Chinese medicine (TCM) in order to assist in the treatment of glioma has raised interest. TCM can improve the clinical symptoms of various types of glioma, reduce the adverse reactions after chemotherapy, enhance immunity, and prolong survival times (Scheck et al., 2006). Current research is therefore focused on finding glioma treatment drugs from natural TCM. Radix scutellariae, the root of the *S. baicalensis* Georgi (Chinese skullcap) plant, has been determined to have numerous pharmacological activities in TCM, including anti-inflammatory effects, which are particularly important, in addition, This TCM has an antiapoptotic effect and is currently employed in treating gliomas, although more research is needed. One of the active ingredients of *S. baicalensis* Georgi that plays a role in TCM is the flavonoid baicalin, which has a significant effect in the treatment of gliomas (Kyo et al., 1998; Lee et al., 2005; Zhang et al., 2014); however, there has been less research on the volatile components of this plant.

Therefore, to examine the effects of the volatile components of *S. baicalensis* on gliomas, we extracted the volatile components of *S. baicalensis* by gas chromatography-mass spectrometry (GC-MS) and then searched and matched the active components related to the onset and development of glioma from the Traditional Chinese Medicine Systems Pharmacology (TCMSP) database. Employing the network pharmacology method, we screened the possible pathways of the involvement of *S. baicalensis* Georgi in the onset and development of glioma and further verified and analyzed the key signal nodes through biological experiments to reveal the potential mechanisms of the volatile components of this plant in the treatment and prevention of gliomas.

## Materials and Methods

### Extraction of Volatile Components from *Scutellaria baicalensis* Geologia by Gas Chromatography-Mass Spectrometry

#### Sample Preparation

We crushed and sipped 30 g of *Scutellaria* and divided the result into three equal parts (10 g each), which we later immersed in hot water for 4 h with the ethanol(81.5°C), methanol(64.7°C), and benzenol (71C), respectively. After passing the resulting liquid through a 0.22-µm ultrafiltration membrane, the filtrate was concentrated to 5 ml using a rotary evaporator and filtered through a 0.22-µm filter membrane into a sample injection bottle, which we then analyzed using GC-MS as detailed below.

#### Gas Chromatography Conditions

We heated an RTX-5MS (30 m × 0.25 mm × 0.25 µm) elastic quartz capillary column using a heating program. The column temperature was raised to 210°C at a rate of 10°C/min, then to 220°C at 1°C/min, and then to 280 °C at 10°C/min. The temperature was then kept steady for 1 min. The total determination time was 20 min, the shunt ratio was 20:1, the inlet temperature was 280°C, the column front pressure was 50 kPa, the injection volume was 1 μL, the carrier gas was high purity nitrogen (99.999%), and the flow rate was 1.1 ml/min.

#### Mass Spectrum Conditions

The settings were as follows: electron ionizationion source; ion source temperature of 200°C; electron energy of 70 eV; connector temperature of 250°C; solvent delay of 3.5 min; scanning range: 50–600 amu; and electron multiplier voltage of 1200 V.

#### Screening with the Prior Data

For the screening, we generated the corresponding compound ion flow chart, based on the mass spectrum of each chromatographic peak fragment figure, the literature review, and the database data. We also checked the mass spectrum data on the base peak’s mass-to-charge ratio and the relative abundance condition as the intuitive comparison. Finally, we determined the crude drug’s chemical composition and its physical and chemical properties and pharmacological action.

### Screening and Identifying the Volatile Active Components of *Scutellaria*

The active components of the GC-MS *S. baicalensis* extracts were screened using the TCMSP database under conditions of oral bioavailability of 30%, druggability of 0.18, and blood-brain barrier (BBB) permeability index of −0.3. We downloaded the chemical structure files corresponding to the active components.

### Construction of the Volatile Chemical Composition Library and Target Prediction

We then determined the specific chemical information for Wogonin (a flavonoid-like chemical compound found in *S. baicalensis*) using the PubChem database. We predicted the target genes using BATMAN-TCM combined with the web-based SwissTargetPrediction tool. Lastly, we selected the target protein name, gene name, UniProt ID, and other information.

### Disease-Related Target Analysis

We searched the DisGeNET NCBI-gene and GeneCards databases using the keyword “glioma” and obtained the target GENE-related data for glioma regulation. We employed UniProt to improve the target’s UniProt ID and gene name.

### Gene Ontology and Kyoto Encyclopedia of Genes and Genomes Enrichment Analysis

We entered the retrieved target genes into the Cytoscape software and employed and Gene Ontology **(**GO) and Kyoto Encyclopedia of Genes and Genomes (KEGG) in the ClueGo plug-in for the biological process analysis and pathway analysis of the TCM volatile components acting on glioma-related targets. The parameters were set as ClueGo:Function, GO and KEGG, and Use GO Term Fusion (p ≤ 0.05).

### Construction of the Volatile Component-Target-Disease Pathway for Traditional Chinese Medicine

As per the established Wogonin and disease-related targets, we screened out the common targets of drugs and diseases and constructed the disease-target regulation network of volatile component action targets and glioma-related diseases of Genuine medicinal materials in Henan using the Search Tool for the Retrieval of Interacting Genes/Proteins (STRING) database. We then inserted the acquired volatile components, key targets of compounds, and main pathways for TCM into Cytoscape software, constructing the glioma-related component-target-main disease pathways using Cytoscape’s Merge and Union functions.

### Experimental Verification of Wogonin on Inflammatory Factors and Apoptotic-Related Proteins in Gliomas

We cultured human U251 cells and treated them with 0, 25, 100, and 200 μM of Wogonin (HPLC ≥ 98%, standard) for 24 h, WOG concentration was based on reference (Wang et al., 2013). we used ELISA to analyze the expression levels of inflammatory factors IL-1, IL-6 and tumor necrosis factor alpha (TNF-α) in the cell supernatant by Western blot analysis (four groups, three indicators in total). We used ELISA to detect the expression of apoptosis-related protein caspase-3/cleaved caspase-3 Bcl-2 Bad.

### Cell Culture

We cultured U251 cells with 10% FBS supplemented DMEM in an incubator set to 375% CO_2_. These cells are obtained from the laboratory. We then grouped the cells as follows: control group (0 μM), 25 μM, 100 μM, and 200 μM.

### ELISA

We used ELISA kit (Wanleibio Co., Ltd.) to detect the expression of TNF-α, IL-1β and IL-6 according to the instructions of the kit.

### Western Blot

1) After the cells were lyzed, the protein in it was extracted, and the concentration of the above-mentioned protein was determined using a measurement kit (Wanleibio Co., Ltd. Shenyang, China).
2) We used polyacrylamide gel to extract the protein of each lysate (20 μg), and then transferred it to a polyvinylidene fluoride membrane (Millipore, Bedford, MA, United States).
3) The above membrane was washed with 0.5% Tween 20 (TBST) in Tris buffered saline for 5 min, and then blocked in 5% skim milk for 1 h.
4) The membranes were incubated with antibodies against caspase3/cleavedcaspase-3, BAD, and Bcl-2 (Wanleibio Co., Ltd. Shenyang, China, 1:2000 dilution) overnight at 4°C.
5) Successively thereafter, the membranes were washed three times with TBST for 5 min each, incubated for 1 h at 37°C with rabbit HRP-conjugated antimouse antibody (1:1,000) (Wanleibio Co., Ltd. Shenyang, China), and washed three times with TBST for 5 min each.
6) We used electrochemiluminescence reagent (Wanleibio Co., Ltd. Shenyang, China) to detect the protein bands. *β*-actin was used to normalize the band density.

### Statistical Methods

The statistical analysis was performed using the SPSS v. 23.0 software package, with the measurement data expressed as (x S). We then calculated the differences between groups using a one-way ANOVA, with *p* < 0.05 indicating statistically significant differences.

## Results

### Extraction of Volatile Components from *Scutellaria baicalensis* by Gas Chromatography-Mass Spectrometry

**FIGURE 1:**
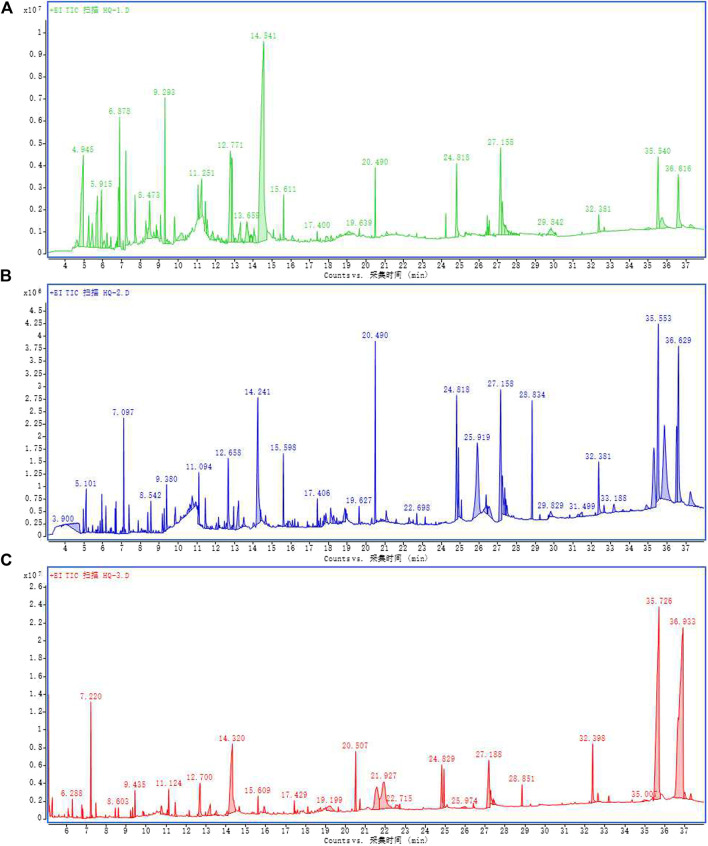
Ion flow diagram of the three gas chromatography-mass spectrometry extraction methods for *S. baicalensis* Georgi by **(A)** ethanol **(B)** methanol, and **(C)** benzyl alcohol.

**FIGURE 2:**
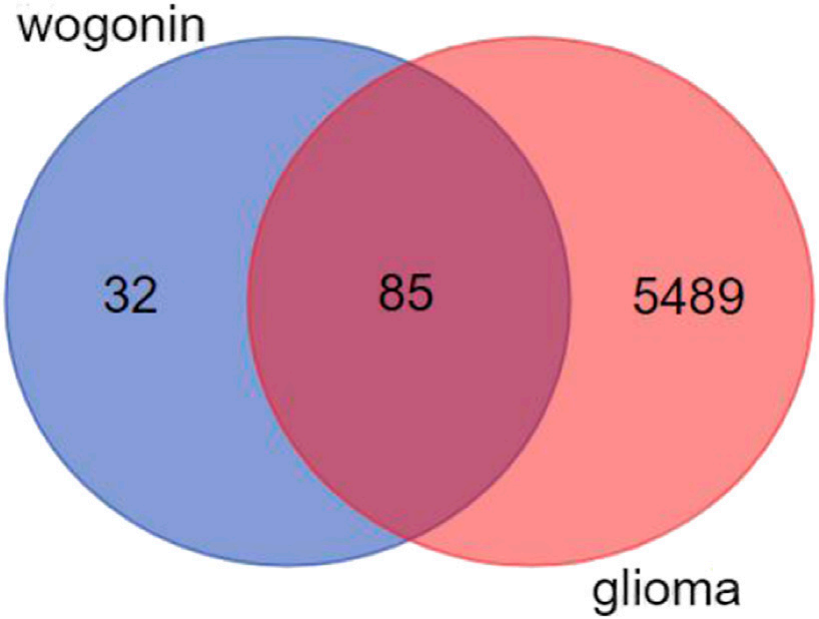
Venn diagram of drug targets and disease proteins.

We used the methanol-ethanol-cresol method to extract the volatile active ingredients of *S. baicalensis* by GC-MS. Figures 1, 2 provide the results of the ion flow diagram. The substances related to the onset and development of gliomas were then searched for and matched on the TCMSP database. We finally determined the volatile active ingredient of Scutellaria, that is Wogonin.

### Network Analysis of Targets

**FIGURE 3:**
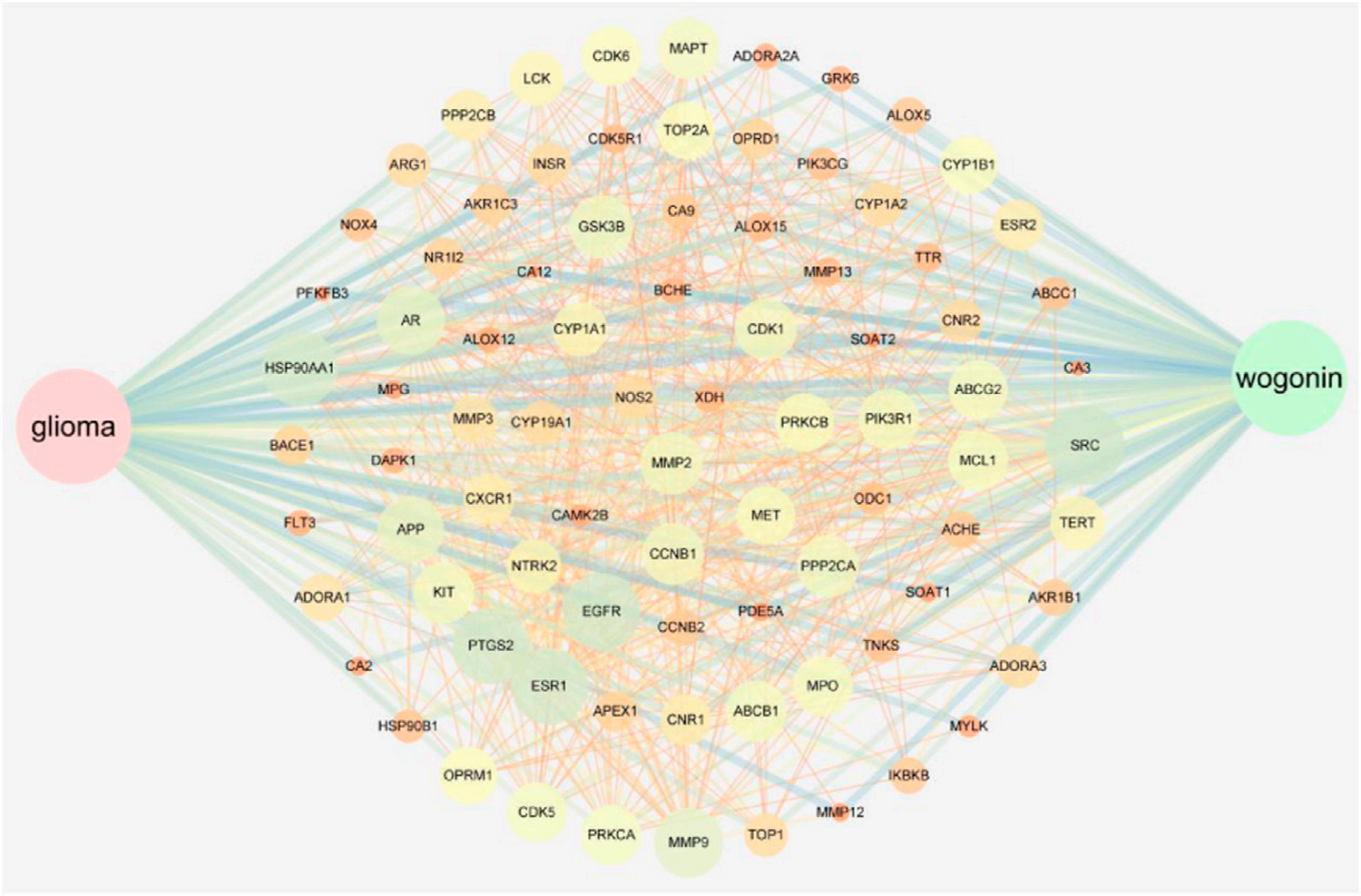
Network analysis of targets compound-target-glioma network.

In total, 5,574 glioma-related targets were retrieved from the GeneCards, DisGeNET database, and NCBI GENE databases. The Bioinformatics Analysis Tool for the Molecular Mechanism of TCM (BATMAN-TCM) combined with the SwissTargetPrediction tool was able to predict the target genes of the volatile components of *S. baicalensis*, obtaining information on 117 common targets, with 85 combined targets for glioma and Wogonin (Figure 2). We employed Cytoscape software to construct the regulatory network of Wogonin acting on glioma (Figure 3).

### Functional Annotation and Kyoto Encyclopedia of Genes and Genomes Pathway Analysis of Glioma Genes

**FIGURE 4:**
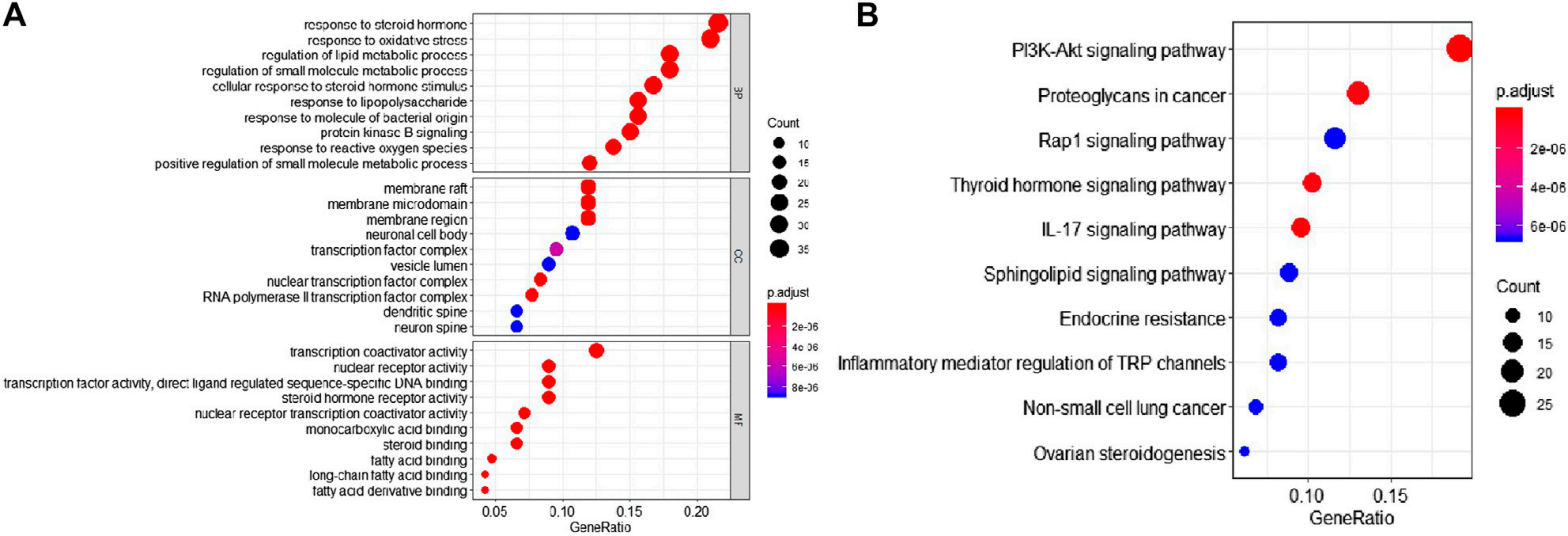
Bioinformatics analysis of drug-disease intersection proteins **(A)** GO annotations **(B)** KEGG annotation.

We employed the clusterProfiler package of R language to perform the GO annotation and KEGG pathway analysis on 85 target genes of Wogonin acting on gliomas. The GO function annotation analysis results showed that these target genes were involved in the binding of phosphatidylinositol phosphokinase-activating chemokines, l-amino acid translocation, amino acid translocation, and reverse translocation (Figure 4A). The KEGG analysis showed that the regulatory pathway included the PI3K-Akt signaling pathway, the Rap1 signaling pathway, and the IL-17 signaling pathway. These results suggest thatWogonin might affect gliomas via these regulatory pathways (Figure 4B).

**FIGURE 5:**
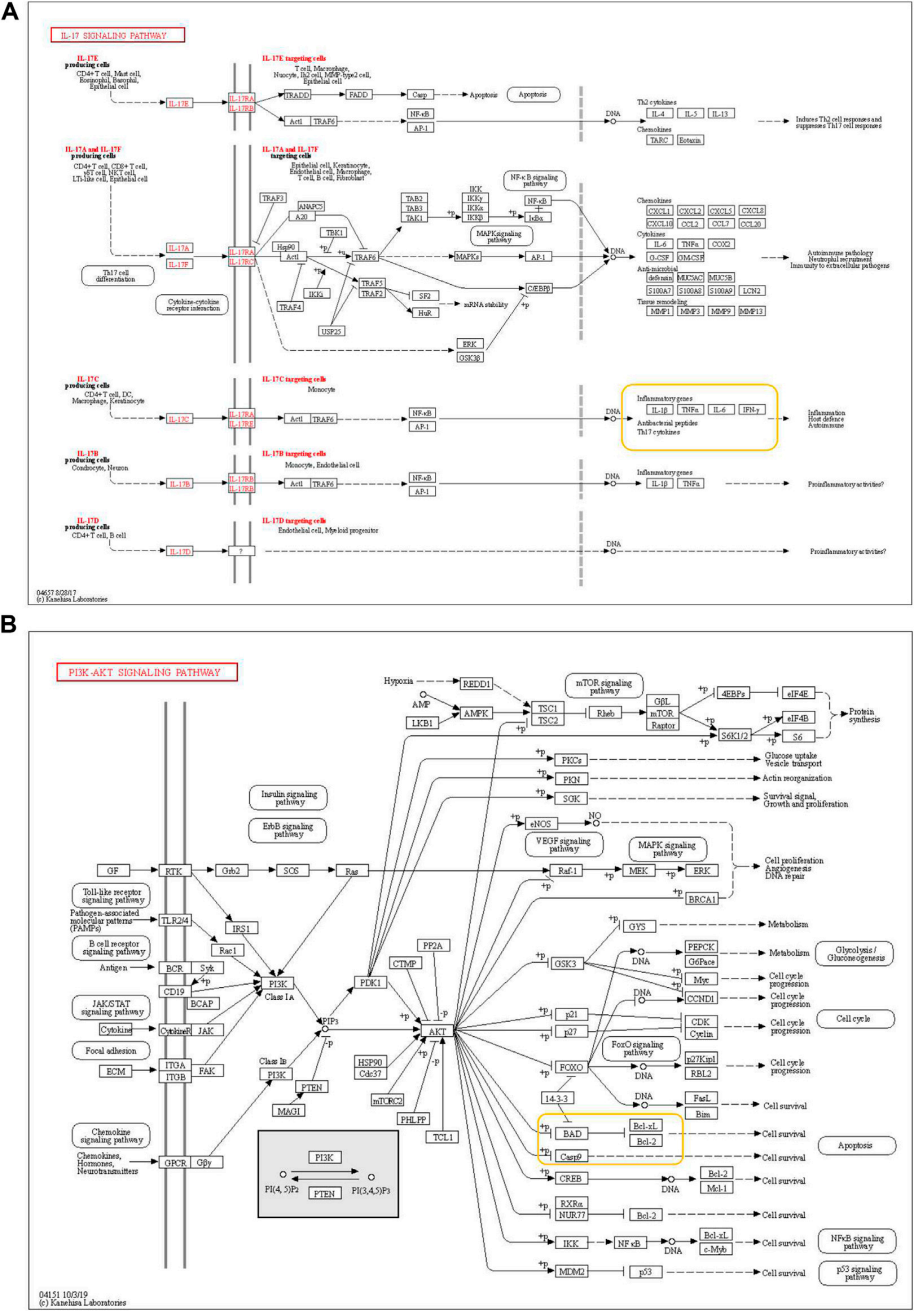
Pi3K-Akt and IL-17 signaling pathway **(A)** PI3K-Akt signaling pathway and **(B)** IL-17 signaling pathway, with key target genes in the yellow box.

The next analysis showed that most of the key target genes are distributed in the PI3K-Akt signaling pathway (an important signaling pathway for tumor apoptosis) and IL-17 signaling pathway (an important inflammatory pathway) (Figure 5). We then selected apoptotic factors such as caspase-3, Bad, and Bcl-2 and inflammatory factors such as IL-1, TNF-α, and IL-6 for further experimental verification.

### Experimental Verification

**FIGURE 6:**
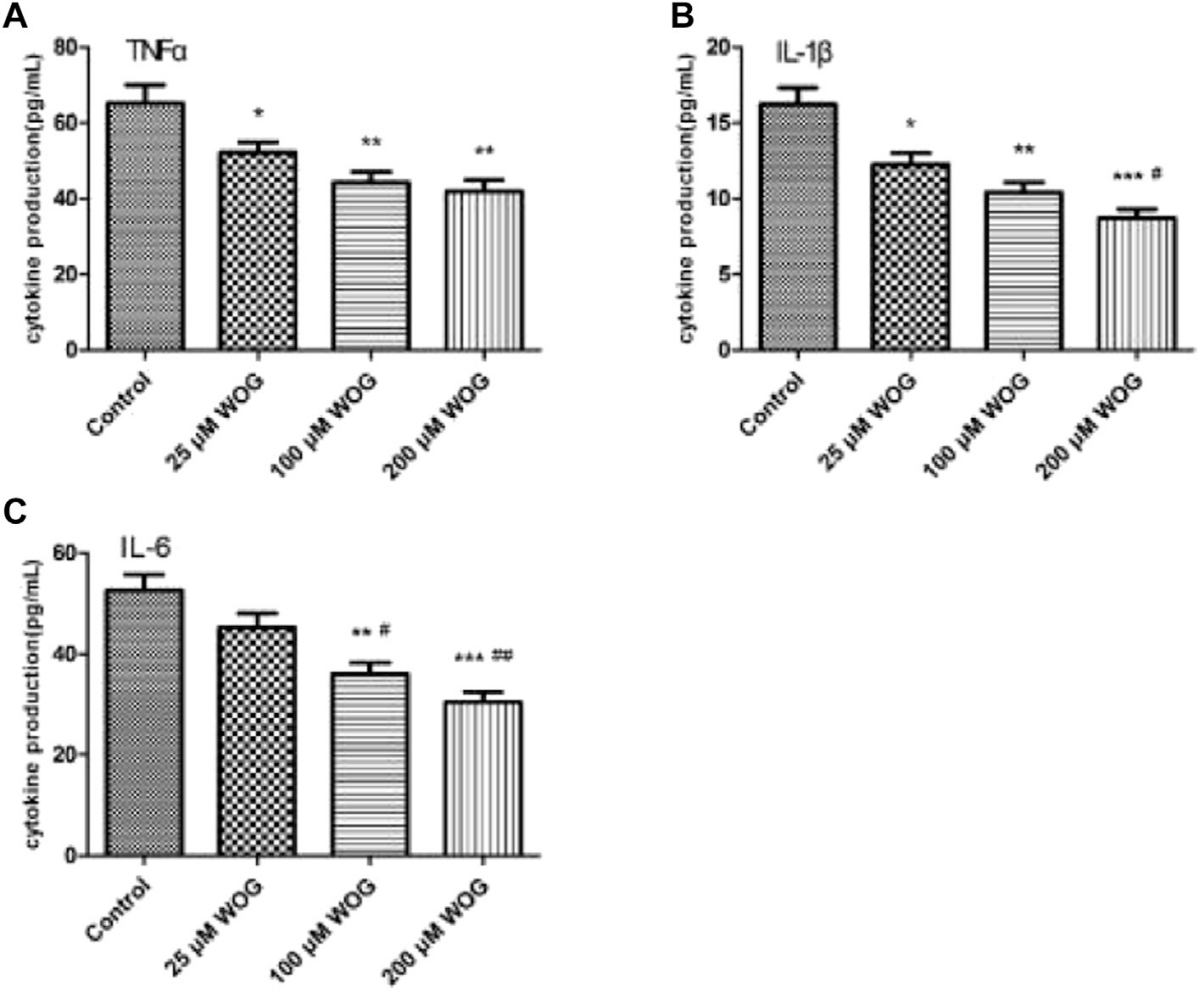
The expression of TNF-α **(A)**, IL-1β **(B)**, and IL-6 **(C)** was inhibited by Wogonin in U251 cells.

The secretion of TNF-α, IL-1β, and IL-6 was detected via ELISA. The groups with Wogonin have showed a decreasing trend in TNF-α, IL-1β, and IL-6 secretion with increased Wogonin concentrations compared with the control group (Table 1). Furthermore, TNF-α downregulation was not significantly dose-dependent on Wogonin (Figure 6A), the inhibitory effect of 25 M of Wogonin on IL-6 was not significant (Figure 6B), and IL-1β downregulation was dose-dependent on Wogonin, with significant differences (Figure 6C).

**TABLE 1:** Wogonin inhibition of the expression of tumor necrosis factor alpha, interleukin-1β, and interleukin-6 in the U251 cells.

Groups	TNF-α	IL-1β	IL-6
Control group	65.173 ± 8.257	16.201 ± 1.921	52.598 ± 5.457
25 μM	52.093 ± 4.917^a^	12.240 ± 1.323^a^	45.182 ± 4.889
100 μM	44.263 ± 4.723^b^	10.393 ± 1.173^b^	36.002 ± 3.874^b^ ^,^ ^d^
200 μM	41.938 ± 4.995^b^	8.727 ± 0.966^c^ ^,^ ^d^	30.417 ± 3.461^c^ ^,^ ^e^

Abbreviations: EPA, eicosapentaenoic acid; IL, interleukin; TNF, tumor necrosis factor.

^a^ *p* < 0.05.

^b^ *p* < 0.01.

^c^ *p* < 0.001 compared with the control group.

^d^ *p* < 0.05.

^e^ *p* < 0.01.

^f^ *p* < 0.001 compared with the 25-μM Wogonin group.

^g^ *p* < 0.05.

&& *p* < 0.01.

&&& *p* < 0.001 compared with the 100-μM Wogonin group.

**FIGURE 7:**
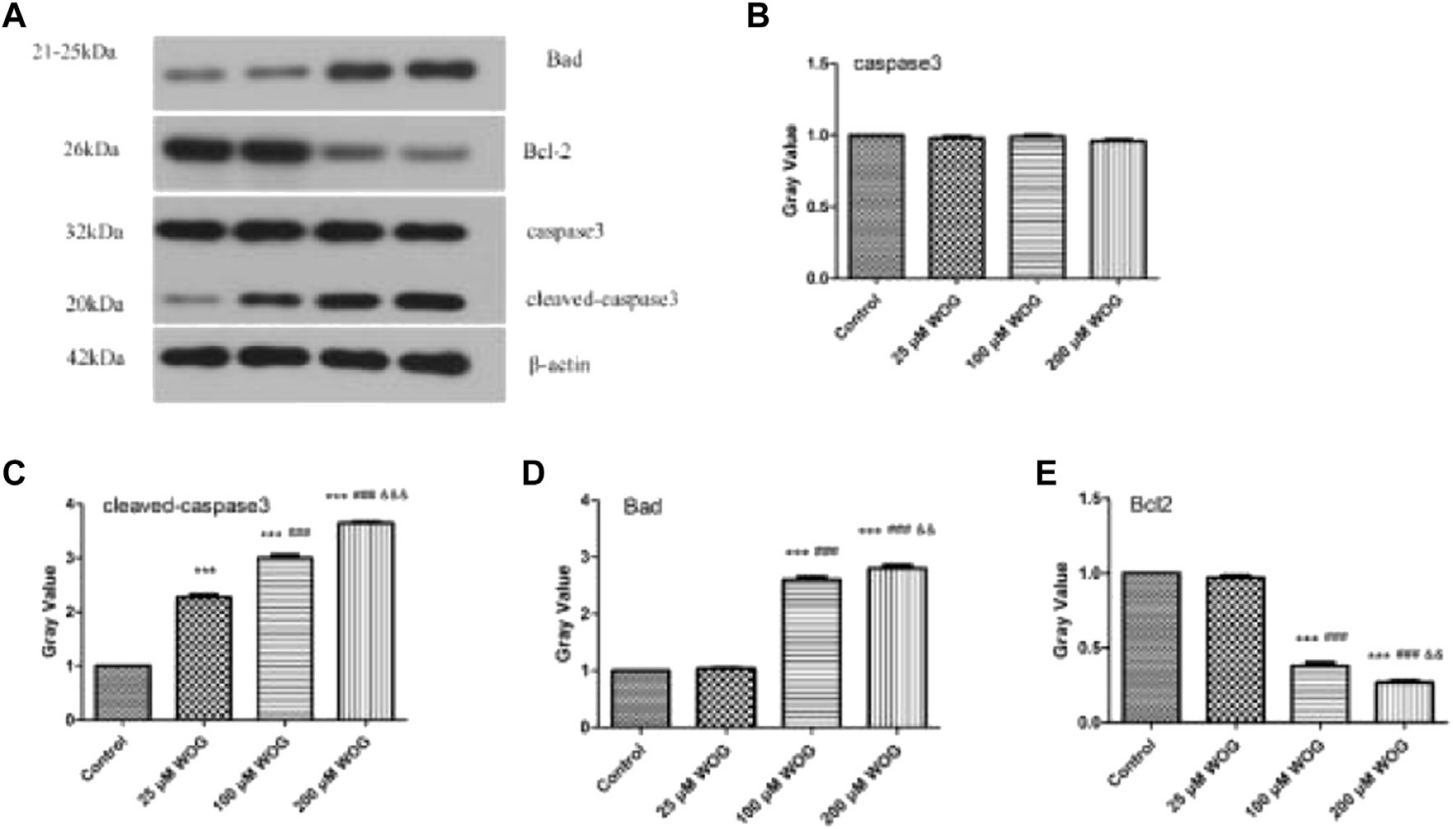
The expression of caspase-3/cleaved-caspase-3, Bad, and Bcl-2 proteins was inhibited by Wogonin in U251 cells **(A)** Western blot analysis **(B–E)** Gray analysis.

We detected the expression of apoptotic regulation genes such as caspase-3/cleaved caspase-3, Bad, and Bcl-2 by Western blotting (Figure 7A). The results showed that the expression of caspase-3 in the experimental group was not significantly different from the control group (Figure 7B), in which cleaved caspase-3 expression was significantly increased and was dose-dependent on Wogonin (Figure 7C). The expression of Bad and Bcl-2 with 25-μM Wogonin has also remained unchanged; however, when the Wogonin dose was increased to 100 μM, the expression of Bad and Bcl-2 was significantly changed (Bad was significantly upregulated, while Bcl-2 was significantly decreased) and was dose-dependent on Wogonin (Figures 7D,E). The relative protein expression of each group was shown in Table 2. In summary, the above results indicate that Wogonin can effectively reduce the apoptosis of glioma cells by regulating the expression of related proteins, which in turn proves the results of the functional enrichment analysis.

**TABLE 2:** The relative expression of caspase-3/cleaved-caspase-3, Bad, and Bcl-2 proteins.

Proteins	Control	25-μM WOG	100-μM WOG	200-μM WOG
Caspase3	1.00 ± 0.03	0.98 ± 0.05	0.99 ± 0.01	0.96 ± 0.07
Cleaved-caspase3	1.00 ± 0.02	2.28 ± 0.04^c^	3.00 ± 0.05^c^ ^,^ ^f^	3.64 ± 0.07^c^ ^,^ ^f^ ^,^ ^i^
Bad	1.00 ± 0.02	1.04 ± 0.02	2.61 ± 0.01^c^ ^,^ ^f^	2.81 ± 0.07^c^ ^,^ ^f^ ^,^ ^h^
Bcl-2	1.00 ± 0.01	0.97 ± 0.05	0.38 ± 0.04^c^ ^,^ ^f^	0.27 ± 0.02^c^ ^,^ ^f^ ^,^ ^h^

^a^ *p* < 0.05.

^b^ *p* < 0.01.

^c^ *p* < 0.001 compared with the control group.

^d^ *p* < 0.05.

^e^ *p* < 0.01.

^f^ *p* < 0.001 compared with the 25-μM Wogonin group.

^g^ *p* < 0.05.

&& *p* < 0.01.

&&& *p* < 0.001 compared with the 100-μM Wogonin group. (n = 3).

## Discussion

In recent years, extracting effective antitumor components with low toxic and adverse effects from TCM and natural plants has become a new line of research. TCM currently employs mostly boiling water processing in extracting the components of hydrothermally soluble substances in TCM materials. The pharmacological effects of non-hydrothermally soluble substances contained in TCM have gradually gained the attention of researchers (Tu, 2011). Other volatile components of TCM are currently being analyzed and applied in the prevention and treatment of tumors (Valentovic, 2018). It has been established that the volatile components of TCM have low molecular weight and strong lipid solubility, and can change the ultrastructure of the biofilm barrier in a variety of ways, including inhibiting P-glycoprotein, so that it can easily pass through the BBB, regulate the permeability of the BBB, andenhance brain targeting and affect neurotransmitter content (Fan et al., 2015). In view of the complexity of the volatile components of TCM, the most critical step in their use is screening the content of the different types of volatile components and classifying their efficacy. In this study, we fully extracted the volatile active components of *S. baicalensis* Georgi (methanol, ethanol, and benzenol) and analyzed the components with GC-MS. We then searched and matched those components related to the onset and development of glioma using the TCMSP database. Lastly, we identified the volatile active component Wogonin by analyzing the results of the GC-MS.

Although TCM has been gaining attention worldwide, understanding the scientific basis of TCM from the molecular and systemic level is still a major challenge for evidence-based medicine (Li et al., 2017; Sidders et al., 2018). Unlike most modern medical approaches, the role of TCM and its compounds in disease prevention and treatment lies in the overall complex community relationship between the numerous components, targets, pathways, and functions (Poornima et al., 2016). Network pharmacology employs biological networks as targets to analyze the predictable and systematic relationship between drug targets and diseases in biological networks (Jain et al., 2018). With the rapid development of bioinformatics and pharmacology, the development of network-based drugs has been recognized as a more cost-effective approach to drug development (Jain et al., 2018).

This study has analyzed the molecular mechanism of *S. baicalensis Georgi* in gliomas by means of network pharmacology. The results revealed 85 targets of Wogonin acting with glioma. The results of the GO annotation and KEGG pathway analysis showed that these target genes were involved in the binding of phosphatidylinositol phosphokinase (to activate chemokines) and l-amino acid translocation (to activate amino acid translocation and thus activate and reverse the transport. The regulatory pathway involves the PI3K-Akt signaling pathway, the Rap1 signaling pathway, and the IL-17 signaling pathway. These results suggest that Wogonin could affect gliomas through the above regulatory pathways. Further research found that important target genes are concentrated in PI3K-Akt and IL-17 signaling pathways. We selected apoptotic factors such as caspase-3, Bad, and Bcl-2 and inflammatory factors such as IL-1β, TNF-α, and IL-6 for further experimental verification.

Glioma is a common and destructive brain tumor that usually occurs in the glial cells of the brain or spine. Immunological studies have established the importance of the tumor microenvironment as a driver of tumor development. Inflammatory mediators such as IL-1β released by monocytes regulate the transcription network needed for malignant cell growth (Tong et al., 2019). The uncontrolled expression of IL-6 in the central nervous system is usually closely related to the onset of neurodegenerative diseases and gliomas (Spooren et al., 2011). TNF-α is an initiator of inflammatory responses and has multipotent pro-inflammatory and neurotoxic effects (Tyrinova et al., 2018). The results of this study showed that 25, 100, 200 μM of Wogonin inhibited the levels of these inflammatory factors in U251 glioma cells, and the IL-1 and IL-6 levels decreased in a dose-dependent manner with the increase in Wogonin concentration.

Wogonin, a monomer component extracted from *S. baicalensis* Georgi, is a flavonoid with a relative molecular weight of 284.26 and a molecular formula of C_16_H_12_O_5_. *In vivo* and *in vitro* analysis found that Wogonin can reduce the growth and metastasis of leukemia, liver cancer, colon cancer, breast cancer, and other malignant tumors (Huynh et al., 2017). Wogonin also strongly hinders the proliferation of U251 and U87 cells in glioma and induces their apoptosis (Tsai et al., 2012). (Wang et al., 2013) extracted Wogonin from *S. baicalensis* and found that Wogonin can inhibit the proliferation of glioma cells and induce G0/G1 stagnation in a dose-dependent manner. The expression of G1-phase cyclin D1 and cyclin-dependent kinases 2 and 4 was significantly decreased, and the cyclin-inhibiting protein P27 was overexpressed. The mechanism of promoting glioma differentiation might be the inhibition of glycogen synthase kinase-3/-light-chain protein pathway (Zhao et al., 2018). suggested that Wogonin could induce the phosphorylation and acetylation of p53, inhibit mouse double minute 2 homolog expression, and enhance p53 stability, thus suggesting Wogonin’s wide range of antitumor effects. Another study on the effects of Wogonin on the proliferation and invasion of U87 glioma cells and its related mechanisms showed that Wogonin has the ability to inhibit U87 cell proliferation and invasion, decrease ezrin protein Bcl-2 and phosphorylated ezrin protein levels, and significantly increase Bax protein expression and the apoptosis index. The authors speculated that Wogonin might inhibit the antiapoptotic factor and promote the apoptosis-inducing factor, thereby inducing glioma cell apoptosis (Zeng and Liu, 2015). Bcl-2 is a classical anti-apoptotic protein in highly homologous proteins and plays an important regulatory role in the process of cell apoptosis (Wang et al., 2018). The downstream factor Bad is a target pro-apoptotic gene that regulates cell apoptosis and survival. Caspase-3 is the central link in the signal transduction process of apoptosis, and the detection of activated caspase-3 can reflect the process of apoptosis (Shen et al., 2016). In this study, we detected the apoptotic factor levels using Western blotting, and the results showed that cleaved caspase-3 expression was significantly increased and was dose-dependent on Wogonin. The expression of Bad and Bcl-2 with 25 μM of Wogonin has remained unchanged; however, when the Wogonin dose was 100 μM, the expression of Bad and Bcl-2 changed significantly (Bad was significantly upregulated, while Bcl-2 was significantly decreased) and was dose-dependent on Wogonin.

In conclusion, we speculate that Wogonin might promote glioma cell apoptosis by upregulating Bad gene expression and cleaved caspase-3 gene activation and by downregulating Bcl-2 expression. Wogonin also inhibits the progression of gliomas by downregulating the secretion of the inflammatory cytokines TNF-α, IL-6, and IL-1β. To provide a theoretical basis for clinical treatment, further in-depth experimental studies are needed to explore the specific molecular mechanisms of Wogonin’s action on gliomas.

## Data Availability

The original contributions presented in the study are included in the article/Supplementary Material, further inquiries can be directed to the corresponding author.
